# Hypoxia-Inducible Factors and the Regulation of Lipid Metabolism

**DOI:** 10.3390/cells8030214

**Published:** 2019-03-03

**Authors:** Ilias Mylonis, George Simos, Efrosyni Paraskeva

**Affiliations:** 1 Laboratory of Biochemistry, Faculty of Medicine, University of Thessaly, BIOPOLIS, 41500 Larissa, Greece; mylonis@med.uth.gr; 2 Gerald Bronfman Department of Oncology, Faculty of Medicine, McGill University, Montreal, QC H4A 3T2, Canada; 3 Laboratory of Physiology, Faculty of Medicine, University of Thessaly, BIOPOLIS, 41500 Larissa, Greece

**Keywords:** hypoxia, HIF, lipids, metabolism, cancer

## Abstract

Oxygen deprivation or hypoxia characterizes a number of serious pathological conditions and elicits a number of adaptive changes that are mainly mediated at the transcriptional level by the family of hypoxia-inducible factors (HIFs). The HIF target gene repertoire includes genes responsible for the regulation of metabolism, oxygen delivery and cell survival. Although the involvement of HIFs in the regulation of carbohydrate metabolism and the switch to anaerobic glycolysis under hypoxia is well established, their role in the control of lipid anabolism and catabolism remains still relatively obscure. Recent evidence indicates that many aspects of lipid metabolism are modified during hypoxia or in tumor cells in a HIF-dependent manner, contributing significantly to the pathogenesis and/or progression of cancer and metabolic disorders. However, direct transcriptional regulation by HIFs has been only demonstrated in relatively few cases, leaving open the exact and isoform-specific mechanisms that underlie HIF-dependency. This review summarizes the evidence for both direct and indirect roles of HIFs in the regulation of genes involved in lipid metabolism as well as the involvement of HIFs in various diseases as demonstrated by studies with transgenic animal models.

## 1. Oxygen Sensing and Hypoxia-Inducible Factor (HIF) Regulation

Insufficient oxygen availability in cells and tissues (hypoxia), a consequence of an imbalance between oxygen supply and metabolic demand, is encountered both physiologically (i.e., during intense exercise or embryogenesis) and in pathological conditions such as cancer, ischemia and metabolism related diseases. Response to hypoxia comprises reduction of oxygen consumption, via metabolic adjustments, and intensification of mechanisms responsible for oxygen transport to cells such as upregulation of erythropoiesis and angiogenesis. These adaptations require extensive reprogramming of gene expression, coordination of which is achieved by the hypoxia-inducible factors (HIFs) [1].

HIFs are heterodimeric transcription factors that consist of an oxygen regulated alpha subunit and a constitutively expressed beta subunit, also known as ARNT (aryl hydrocarbon receptor nuclear translocator), both members of the basic helix-loop-helix (bHLH) proteins of the PER-ARNT-SIM (PAS) DNA binding protein family. The active heterodimer binds to hypoxia-response elements (HREs) on the promoter or enhancer regions of target genes, causing their transcriptional activation [2]. Three HIF-α isoforms have been identified to date. HIF-1α is expressed ubiquitously in cells and tissues, while, HIF-2α (termed also EPAS1) is tissue specific [3,4]. The third and least studied HIF-α isoform, HIF-3α, exists in multiple splice variants most of which act as dominant-negative regulators of HIF activity [5,6].

**Figure 1:**
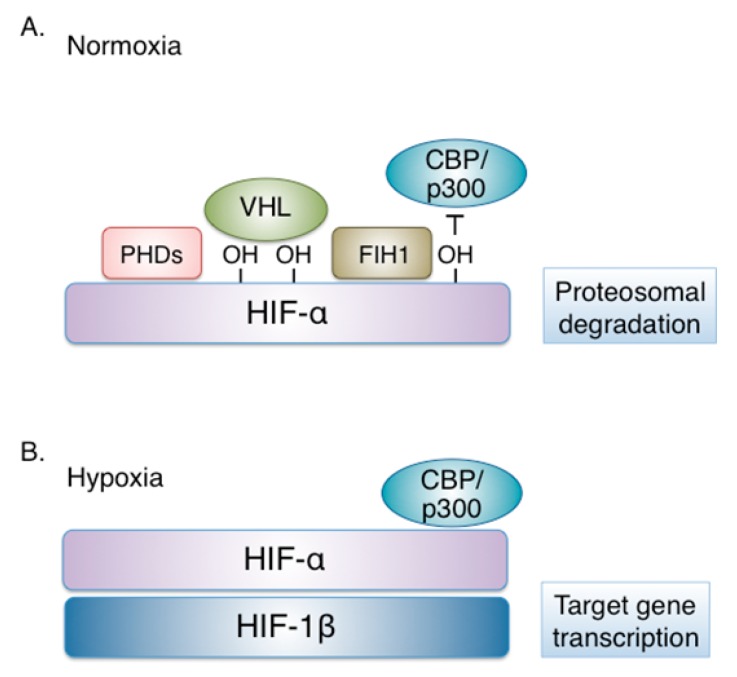
Regulation of hypoxia-inducible factor (HIF) by oxygen. (**A**) Under physiological oxygen concentration (Normoxia), HIF-α isoforms are modified by oxygen-dependent prolyl-hydroxylases (PHDs), recognized by the von Hippel–Lindau (VHL) tumor suppressor protein, ubiquitinated and targeted to the proteasome for degradation. In addition, HIF-α modification by FIH (factor-inhibiting HIF), an oxygen-sensitive asparaginyl hydroxylase, disrupts interaction with the transcriptional co-activators p300/CBP and impairs residual HIF transcriptional activity. (**B**) When oxygen becomes limited (Hypoxia), PHDs and FIH are inactive. The non-hydroxylated HIF-α is stable and dimerizes with HIF-1β. The HIF heterodimer interacts with p300/CBP and activates the transcription of HIF target genes.

Under physiological oxygen conditions, HIF-α isoforms are constantly produced and destroyed in a process that involves hydroxylation at two proline residues within a conserved HIF-α region termed oxygen dependent degradation domain (ODDD). This modification is catalyzed by three prolyl-hydroxylases (PHDs), enzymes that act as “oxygen sensors” in the cell, as their catalytic activity requires oxygen as a substrate. Following hydroxylation, HIF-α is recognized by the von Hippel–Lindau (VHL) tumor suppressor protein, an E3 ubiquitin ligase complex member, resulting to HIF-1α ubiquitination, targeting to the proteasome and degradation [7] (Figure 1). Another oxygen-sensitive enzyme, the asparaginyl hydroxylase FIH (factor-inhibiting HIF) modifies HIF-α subunits at the C-terminal transactivation domain and disrupts the interaction between HIF-α and the transcriptional co-activators p300/CBP thereby impairing residual HIF transcriptional activity [8].

**Figure 2:**
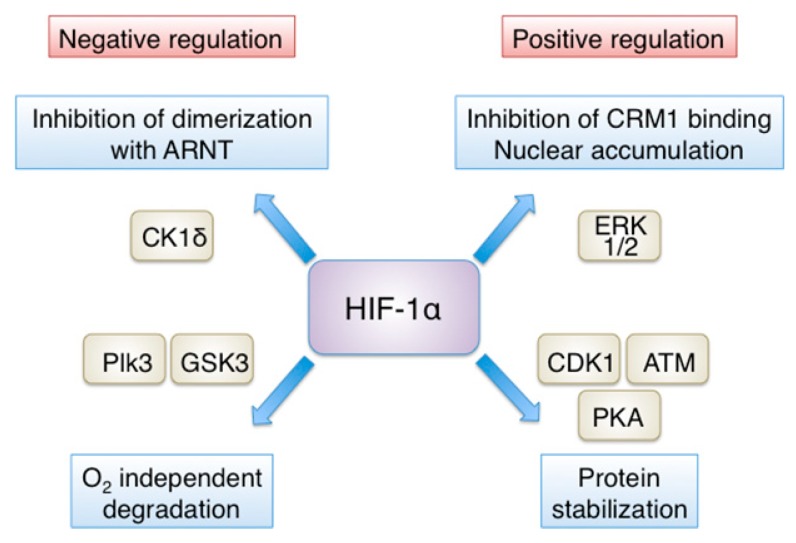
Positive and negative regulation of HIF-1α by phosphorylation. Direct phosphorylation by several kinases is important for HIF-1α regulation. Positive regulation: ERK1/2-dependent phosphorylation inhibits binding of the exportin CRM1 and promotes nuclear accumulation of HIF-1α, while phosphorylation by ATM, CDK1 or PKA inhibits HIF-1α degradation. Negative regulation: phosphorylation by casein CK1δ impairs HIF-1α association with ARNT and thus, decreases HIF-1 transcriptional activity, while phosphorylation by GSK3 or Plk3 results in VHL-independent degradation of HIF-1α. See text for details and references.

In addition to oxygen tension, HIF-1 expression and activity are also controlled by oxygen-independent mechanisms regulating gene transcription, mRNA translation, protein–protein interactions and post-translational modification of the HIF-1α subunit. Transcriptional upregulation of the HIF-1α gene (*HIF1A*) in response to inflammation is achieved in a NF-κB-dependent manner [9,10,11]. Transcription of *HIF1A* also involves STAT3 (signal transducer and activator of transcription 3) [12] and Sp1 [13]. Moreover, activation of the PI-3K/AKT pathway by growth factors leads to increased HIF-1α mRNA and protein synthesis (reviewed in [2]). HIF-1α is also regulated through its association with other proteins. To mention only few examples, HIF-1α interaction with the molecular chaperone HSP90 results in its stabilization, whereas binding to RACK1, has the opposite effect [14,15,16]. Post-translationally, in addition to hydroxylation, HIF-1α is subject to SUMOylation [17,18,19,20], acetylation [21,22], deacetylation [23] and S-nitrosylation [24], although the impact of these modifications on HIF-1α stability and/or activity has not yet been adequately clarified. In contrast, direct phosphorylation by several kinases is important for HIF-1α regulation and is extensively studied (reviewed in [25]) (Figure 2).

Phosphorylation by GSK3 (glycogen synthase kinase 3) at three residues within the N-terminal transactivation domain causes degradation of HIF-1α in a VHL-independent manner [26]. A similar role has also been proposed for Plk3 (Polo-like kinase 3)-mediated phosphorylation of HIF-1α [27]. On the other hand, direct modifications of HIF-1α by ATM [28], CDK1 [29] or PKA [30] have been shown that stabilize HIF-1α by inhibiting its degradation. Downstream of its stabilization, transcriptional activity of HIF-1α also depends of its efficient accumulation inside the nucleus, a process regulated by ERK1/2-dependent phosphorylation. Translocation of HIF-1α inside the nucleus appears to be constitutive and is mediated by multiple import receptors; the importin α/β family, which recognize a nuclear localization signal (NLS) at the C-terminal domain of HIF-1α [31,32,33], as well as importins 4/7, which interact with the N-terminal part of HIF-1α [34]. However, CRM1-dependent nuclear export of HIF-1α depends in its modification by ERK1/2, which phosphorylates HIF-1α at sites adjacent to an atypical hydrophobic nuclear export signal (NES), thereby preventing CRM1 binding and increasing the nuclear concentration and activity of HIF-1α [35,36]. Inhibition of ERK-mediated phosphorylation of HIF-1α tips the balance in favor of nuclear export and cytoplasmic localization of a major pool of HIF-1α, which is bound by mortalin and targeted to the mitochondrial surface, where it participates to the formation of an anti-apoptotic complex [37]. 

Finally, phosphorylation by CK1δ (casein kinase 1δ) within the PAS domain impairs HIF-1α association with ARNT, hinders the formation of a functional heterodimer and thus, decreases HIF-1 transcriptional activity [38]. Interestingly, the association between HIF-1α and ARNT can also be inhibited by interaction of HIF-1α with MgcRacGAP (male germ cell RacGTPase Activating Protein) in cancer cells [39,40] or after treatment of human bronchial smooth muscle cells with the proinflammatory factor TNF-α [11]. Much less is known regarding the oxygen-independent mechanisms and post-translational modifications that regulate HIF-2α. The few examples include deacetylation by Sirt1, which enhances HIF-2α activity [41] and phosphorylation by CK1δ, which, in contrast to HIF-1α, promotes HIF-2α nuclear activity [42]. The crosstalk between the signaling pathways, that result to modification and regulation of the HIF-α isoforms, with those controlling metabolic homeostasis may ultimately define the exact role of HIFs in the metabolic adaptation of cells to hypoxia.

## 2. The Involvement of HIFs in the Regulation of Lipid Metabolism

When oxygen is sparse, cells adapt to hypoxia by reprogramming the expression of a number of genes involved in energy metabolism. The role of HIF-1 in the activation of genes encoding for proteins involved in carbohydrate metabolism has long been established (reviewed in [43,44]). HIF-1 not only promotes glucose uptake by activating the transcription of transporters GLUT1 and GLUT3, but also enhances anaerobic energy production, as it upregulates most of the glycolytic enzymes (including HK1/2, ENO1, PGK1 and PKM2) and proteins that facilitate the synthesis and excretion of lactate (LDH and MCT4). Moreover, in order to reduce mitochondrial function for decreasing consumption of oxygen and ROS production, HIF-1 stimulates the expression of pyruvate dehydrogenase kinase (PDK1) and BNIP3 [45,46,47]. PDK inhibits the pyruvate dehydrogenase complex and blocks the conversion of pyruvate, the glycolytic end product, to acetyl-CoA, which normally feeds into TCA cycle by producing citrate. Therefore, the flow of pyruvate into the mitochondria is decreased, fueling the production of lactate by LDH in the cytoplasm. On the other hand, BNIP3 triggers mitochondrial autophagy, further reducing mitochondrial metabolic processes. 

**Figure 3:**
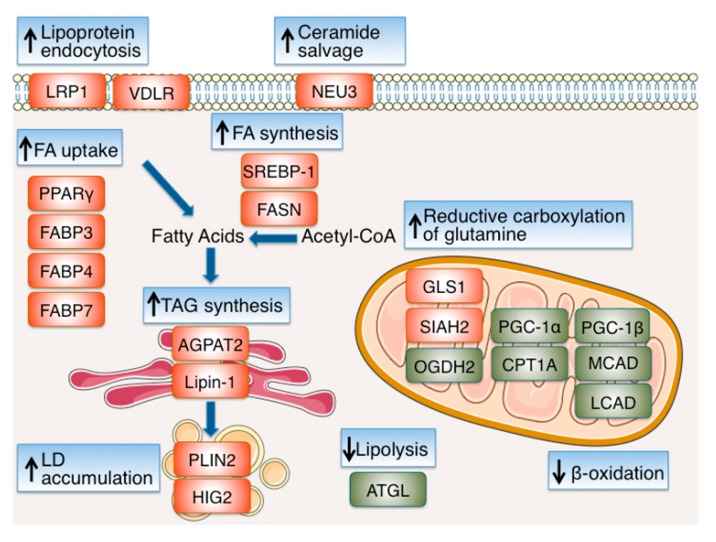
Reprogramming of lipid metabolism under hypoxia. Hypoxia enhances lipogenesis by HIF-dependent modulation of proteins involved in fatty acid (FA) uptake, synthesis, storage and usage. Uptake of extracellular FA is promoted under hypoxia by activation of the transcription factor PPARγ and the increased expression of FABPs 3, 4 and 7. Endocytosis of lipoproteins is enhanced by the upregulation of LRP1 and VLDLR, while ceramide levels are increased by upregulation of NEU3. To maintain de novo FA synthesis under hypoxia, preservation of citrate levels and synthesis of acetyl-CoA is achieved by stimulation of reductive glutamine metabolism, mediated, at least in part, by induction of GLS1 and proteolysis of the OGDH2 subunit of the α-ketoglutarate dehydrogenase complex (αKGDH) by SIAH2. Adequate FA supply is further supported by activation of SREBP-1, which in turn upregulates the expression of FASN. To avoid lipotoxicity and/or replete lipid stores, FAs are converted to neutral triacylglycerols (TAGs), which are stored in lipid droplets (LDs). Formation of LDs under hypoxia is favored by the upregulation of the TAG biosynthesis pathway enzymes AGPAT2 and lipin-1, and the LD membrane proteins PLIN2 and HIG2. Finally, lipid accumulation under hypoxia is additionally supported by the inhibition of β-oxidation through downregulation of PGC-1α, CPT1A, PGC-1β, MCAD and LCAD. The proteins upregulated or activated under hypoxia are shown in red and the proteins downregulated or inhibited under hypoxia are shown in green. See text for details and references.

Despite the extensive literature on HIF-dependent regulation of carbohydrate metabolism, the effects of hypoxia and HIFs on lipid metabolism have only recently become the focus of closer examination (Figure 3). Fatty acids (FAs), provided either by exogenous FA uptake or de novo synthesis, are used as substrates for oxidation and energy production, membrane synthesis, energy storage in form of triacylglycerols (TAGs) and production of signaling molecules and, therefore, are essential for cell survival and proliferation both under normoxia and hypoxia. However, as FA oxidation takes place inside mitochondria and requires oxygen, FA metabolism has to be modified under hypoxia in order to serve mainly processes other than energy production. Furthermore, as conversion of glucose into citrate—the major source of cytoplasmic acetyl-CoA and FA precursor—is prohibited under hypoxia due to the inhibition of the TCA cycle, alternative sources of FA precursors have to be exploited. In tumor cells, which usually have to grow in a hypoxic microenvironment, these hypoxia-mediated changes in lipid metabolism are especially important in order to maintain the high proliferation rate that characterizes cancer cells.

Uptake of extracellular FA and TAG synthesis are promoted under hypoxia by transcription factor PPARγ, the gene of which is a directly activated by HIF-1 [48]. Extracellular FA influx and lipogenesis under hypoxia are also enhanced via HIF-1-mediated induction of the expression of FABP (fatty acid binding protein) 3 and 7 in cancer cells [49] and FABP4 in primary mouse hepatocytes [50]. In addition, HIF-1 can promote the endocytosis of lipoproteins, by upregulating the expression of low-density lipoprotein receptor–related protein (LRP1), the receptor that internalizes LDL in vascular smooth muscle cells [51], as well as the expression of VLDL receptor (VLDLR) in cardiomyocytes [52]. 

To also maintain de novo FA synthesis under hypoxia, production of FA precursors is supported in human renal cell carcinoma (RCC) as well as other cancer cells through HIF-dependent stimulation of reductive glutamine metabolism [53,54]. This proceeds via conversion of glutamine to α-ketoglutarate and its subsequent reductive carboxylation that produces citrate, in a reversion of the TCA cycle reaction catalyzed by IDH (isocitrate dehydrogenase). This may be an indirect result of the HIF-mediated decrease of intracellular citrate levels (due to upregulation of PDK1) but IDH1 or 2 may also actively contribute to the preservation of citrate levels under hypoxia [55,56,57]. Moreover, HIF-1 increases the amount of α-ketoglutarate, which can be used as substrate for citrate synthesis and FA/lipid production, by inducing the expression of GLS1 (glutaminase 1) [58], as well as, by inducing the E3 ubiquitin ligase SIAH2, which in turn mediates the proteolysis of the E1 subunit (OGDH2) of the α-ketoglutarate dehydrogenase complex (αKGDH) [57]. Adequate FA supply is further supported by Akt- and HIF-1-dependent activation of SREBP-1, which in turn upregulates the expression of FASN (fatty acid synthase), an essential lipogenic enzyme, the activity of which is correlated with cancer progression and hypoxia induced chemoresistance [59]. 

As FA catabolism is impaired under hypoxia, an excess of intracellularly accumulated free FAs could cause lipotoxicity. To avoid this, cells can convert FAs to neutral TAGs, that are stored in lipid droplets (LDs) and can serve as the main form of energy depots [60,61]. Two enzymes of the TAG biosynthesis pathway, AGPAT2 (acylglycerol-3-phosphate acyltransferase 2) [62] and lipin-1 [63], have been shown to mediate hypoxia-induced LD accumulation. AGPAT2, or else LPAATβ (lysophosphatidic acid acyltransferase β), catalyzes the conversion of lysophosphatidic acid (LPA) to phosphatidic acid (PA). Interestingly *AGPAT2*, which is a direct target of HIF-1 [62], is one of the genes mutated in patients with congenital generalized lipodystrophy, and is upregulated in biopsies from cancer patients. Likewise, HIF-1 also directly upregulates the expression of lipin-1, a phosphatidic acid (PA) phosphatase that catalyzes the conversion of PA to diacylglycerol (DAG) in TAG synthesis [63]. AGPAT2 and lipin-1 upregulation is necessary for LD accumulation and increased viability and chemoresistance under hypoxia [62,63,64]. The importance of the hypoxic upregulation of AGPAT2 and lipin-1 may extend beyond the formation of lipid droplets. The products of their catalytic activity LPA and PA can either be used as precursors of TAGs or as precursors for the synthesis of phospholipids, which are important blocks for new membrane formation [61]. Formation of lipid droplets under hypoxia is further favored by the hypoxic induction of essential constituents of LD membranes. Stimulation of the LD coat protein adipophilin/perilipin 2 (PLIN2) expression by HIF-2 promotes RCC lipid storage, ER homeostasis and viability [65], and the induction of HIG2/HILPDA (Hypoxia-inducible protein 2/hypoxia-inducible lipid droplet associated) by HIF-1 increases lipid accumulation in both cancer and normal cells [66,67]. Furthermore, HIG2 upregulation under hypoxia inhibits the adipose triglyceride lipase (ATGL) and impairs intracellular lipolysis in various cancer cells [68].

Finally, lipid accumulation under hypoxia is additionally supported by the inhibition of enzymes involved in fatty acid degradation. Under low oxygen concentration, fatty acid β-oxidation is actively reduced by HIF-1- and HIF-2-dependent downregulation of the transcriptional coactivator of β-oxidation enzyme PGC-1α (proliferator-activated receptor-γ coactivator-1α) [69] and carnitine palmitoyltransferase 1A (*CPT1A*), the limiting component of mitochondrial fatty acid transport, in both hepatoma and RCC cells [69,70] as well as by the HIF-1-mediated decreased expression of MCAD and LCAD (medium- and long-chain acyl-CoA dehydrogenases) in hepatoma cells, which depends on the hypoxic inhibition of PGC-1β, a transcription factor involved in mitochondrial regulation [71]. As HIFs have not been shown to possess intrinsic transcription repressor activity, downregulation of these enzymes may be mediated by the action of HIF-1 target genes that remain, in most cases, to be identified. In summary, hypoxia overall causes enhanced lipogenesis by HIF-dependent induction of genes involved in FA uptake, synthesis and storage (Table 1). Importantly, as discussed below, induction of these genes and subsequent lipid accumulation are indispensable for cancer cell proliferation under hypoxia.

## 3. HIF-Dependent Regulation of Lipid Metabolism and Cancer Cell Proliferation

Hypoxia develops in tumors as a consequence of the high proliferation rate of cancer cells and aberrant angiogenesis. Activation of the hypoxia response pathway helps cancer cells to adapt and survive by affecting multiple metabolic pathways [72]. Enhanced esterification of free FAs to neutral TAGs and storage in expanded LDs, protects cancer cells from lipotoxicity [73]. In addition, segregation of free FAs in LDs protects solid tumor cancer cells that are exposed to intermittent hypoxia from the lethal formation of free radicals during cycles of hypoxia and reoxygenation [49,65,74,75].

Besides their role in sequestering potential harmful FAs, LDs serve as energy stores and reservoirs of building blocks for the production of the essential sterol esters and phospholipids required in proliferating cells for the biogenesis of new membranes (reviewed in [76]). A connection between HIF-induced TG synthesis and cell proliferation is supported by metabolic profiling analysis of cancer cells kept under hypoxia, which has shown that the concentration of TAGs and derivative phospholipids PC and PE is substantially increased in a HIF-1α-dependent manner [77]. It has to be pointed out that, in many cancer types, silencing of HIFs or interfering with the expression of its target genes required for lipid accumulation, results in reduction of proliferation potential and chemoresistance under hypoxia [49,55,57,59,62,63,65,68,70,78,79]. Moreover, the overexpression of HIF-regulated genes involved in lipid metabolism has been correlated with malignant subtypes of human cancers or poor patient prognosis [70,79]. Intervening with HIF-dependent reprogramming of lipid metabolism can indeed suppress effectively cancer cell proliferation. Systemic administration of glutaminase inhibitors suppressed the growth of RCC cells as xenografts in mice [54], while recent studies have shown that modulation of HIF-1α phosphorylation can regulate LD accumulation and cancer cell growth, specifically under hypoxia [64,80]. Modification of HIF-1α by CK1δ reduced induction of lipin-1 and restricted lipid droplet formation and cell proliferation under hypoxia in a HIF-1 and lipin-1-dependent manner [64]. In addition, inhibition of ERK-mediated phosphorylation of HIF-1α by transduced recombinant HIF-1α-derived peptides abolished induction of lipin-1 expression, reduced lipid droplet accumulation and triggered apoptosis in cancer cells grown under hypoxia [80]. 

## 4. HIF-Dependent Regulation of Lipid Metabolism in Obesity and Metabolic Syndrome

HIF-dependent regulation of lipid metabolism in response to hypoxia, or other stimuli including diet, has been implicated in disorders affecting organs involved in lipid processing and storage, such as the adipose tissue and the liver. In obesity, enlargement of adipocytes beyond the oxygen diffusion limit and their distancing from the vasculature, leads to the development of local hypoxia (reviewed in [81]). Accordingly, visceral adipose tissue from obese human subjects is characterized by increased expression of HIF-1α [82]. Hypoxia has been shown to increase liver lipid contents via induction of HIFs in mice [69] and hepatocellular carcinoma cells [63], while a theoretical model of hepatic lipid accumulation, suggests that hypoxia is contributing to lipid accumulation and steatosis [83]. In addition, several studies have shown that liver HIF stabilization after hepatocyte-specific VHL deletion increased liver lipid accumulation [45,84,85]. In one case, liver specific overexpression of constitutively active forms of both HIF-1α and HIF-2α was required to phenocopy the VHL deletion suggesting that both isoforms are involved in the accumulation of lipids in the liver [45]. On the other hand, experiments in which HIF-1α, HIF-2α or both isoforms were deleted concomitantly with VHL indicated that liver lipid accumulation was mediated predominantly via activation of HIF-2α [84,85]. A number of animal studies based on the deletion or overexpression of HIFs or other components of the hypoxia-response network, suggest that HIF activation can be either beneficial or detrimental in terms of metabolic disease. As this subject has recently been reviewed [86], only a brief overview will be presented.

### 4.1. HIFs as Suppressors of Obesity

A number of studies have shown that obesity is increased by inhibition of HIFs and decreased by HIF activation. In an in vitro adipocyte differentiation study, hypoxia inhibited adipogenesis via HIF-1-dependent upregulation of DEC1/Stra13 and subsequent repression of PPARγ2 expression [87]. Accordingly, transgenic mice overexpressing an adipose tissue-selective dominant negative HIF-1α mutant that decreased HIF-1 activity, developed increased obesity after high-fat diet treatment and accumulated enlarged adipocyte LDs [88]. Similar phenotypes were also observed after HIF activation in adipose tissue specific *PHD2* knockout (KO) mice [89] or global *FIH* KO mice [90], which in both cases protected from high-fat diet-induced obesity. Interestingly, the effects of neuron-specific *FIH* knockouts resembled those of the global null mutants, suggesting that the nervous system is implicated in the FIH-driven regulation of metabolism [90]. 

### 4.2. HIFs as Promoters of Obesity

In contrast to the above, there have been studies showing that HIF activation induces obesity. Adipocyte specific KO of *HIF1A* [82,91,92,93], inhibition of HIF-1 by acriflavine [91] or PX-478 [94], or adipocyte specific *ARNT* KO [93,95] decreased obesity and insulin resistance in mice fed with high-fat diet. In agreement, adipose specific ablation of the PHD2 gene caused HIF-1-dependent reduction of lipolysis and enhanced adiposity in mice [96]. This effect can be correlated with the capacity of HIF-1 to downregulate FA oxidation in adipose tissue [82]. Resistance of mice with depletion of adipocyte HIF-1α to insulin has also been linked to the downregulation of adiponectin expression via HIF-1-mediated regulation of the SOCS3-STAT3 signal transduction pathway [91].

### 4.3. HIFs and Non-Alcoholic Fatty Liver Disease (NAFLD)

Hypoxia and obesity are also linked to liver diseases, such as non-alcoholic fatty liver disease (NAFLD), characterized by inflammation, fibrosis and steatosis. Diet, adipokines and stress are significant contributing factors in NALFD [97]. Although the causality and molecular mechanisms that underlie NAFLD are not completely understood, the development of hypoxia in the liver is implicated in the pathogenesis of the disease. Hepatocyte-specific HIF-1 activation has been shown to promote alcohol-induced hepatomegaly and hepatic lipid accumulation, while hepatocyte-specific deletion of HIF-1α protected mice from alcohol- and lipopolysaccharide (LPS)-induced liver damage, hepatomegaly and lipid accumulation [98]. In a contrasting study, hepatocyte-specific HIF-1α-null mice exposed to ethanol-containing liquid diet exhibited enhanced accumulation of lipids in the liver, via inactivation of the HIF-1-regulated transcriptional repressor DEC1 [99]. Similarly, HIF-1α liver KO enhanced lipid accumulation in choline deprivation-induced NAFLD [100]. Interestingly in this case, liver lipid accumulation was inhibited by overexpression of lipin-1, the direct target of HIF-1 mediating TAG biosynthesis. This was attributed to a non-catalytic nuclear function of lipin-1 and regulation of the PPARα target genes controlling peroxisomal fatty acid oxidation [100]. Finally, it appears that extrahepatic expression of HIFs may also affect liver lipid metabolism. A recent study has shown that mice with intestine-specific disruption of HIF-2α had substantially lower high-fat-diet-induced hepatic steatosis and obesity compared to control animals [101]. This effect was also reproduced when mice were treated with PT2385, a HIF-2α-specific inhibitor. Subsequent analysis suggested that hepatic steatosis developed as a result of decreased ceramide production in the intestine, a process involving a direct gene target of HIF-2, Neu3 (neuraminidase 3) [101]. 

In conclusion, the animal studies that have investigated the involvement of HIFs in obesity and other metabolic disorders are often conflicting and do not clarify their exact role in the dysregulation of metabolism that contributes to the onset of these disorders. These discrepancies may reflect differences in the genetic background, age and diet of the mice used in these studies or the level of inhibition of HIF activity achieved with the different genetic or pharmaceutical approaches. In addition, they may also result from the complexity of systemic metabolic regulation in combination with the multifaceted roles of HIFs in cellular functions that extend further than lipid metabolism.

## 5. HIF-Dependent Regulation of Lipid Metabolism in Cardiovascular Disease

Deregulation of the adipose tissue function and ectopic lipid accumulation is a primary factor for the development of cardiovascular disease. A number of studies indicate that many of the HIF-target genes involved in lipid metabolism can contribute to cardiovascular pathogenesis. Upregulation of LRP1 by HIF-1 contributes to the deposition of lipids in atherosclerotic plaques in human vascular smooth muscle cells, while vascular cell LRP1 and HIF-1α co-localize in immunohistochemical samples of human advanced atherosclerotic plaques [51]. Another HIF-1 target gene, HIG2/Hilpda, stimulates lesion formation and development of atherosclerosis, as the expression of various atherosclerotic pathogenic markers was decreased by conditional Hilpda KO in macrophages of ApoE-/- mice [67]. This is in line with older in vitro studies showing hypoxia-dependent formation of cytosolic lipid LDs in macrophages [102]. Concerning the direct effects of hypoxia on cardiac function, experiments with ventricular HIF-1α KO mice have shown that HIF-1-induced PPARγ activation contributes to metabolic reprogramming and development of contractile dysfunction under pathological stress [48]. Similarly, VHL-null hearts, in which HIFs were activated, developed a number of features associated with human heart failure, including lipid accumulation, myofibril rarefaction, altered nuclear morphology, myocyte loss, and fibrosis, resulting in premature death [103]. These pathogenic features were prevented by the simultaneous cardiac ablation of both VHL and HIF-1α, strongly suggesting the involvement of HIF-1. Interestingly, deletion of VHL specifically in mice adipocytes also caused the development of lethal cardiac hypertrophy, which was, however rescued by genetic deletion of HIF-2α but not HIF-1α [104]. In contrast to the harmful effects of VHL deletion, inhibition of PHDs that also leads to HIF activation has been suggested to play a protective role in cardiovascular disease. In atherosclerotic mice due to LDLR (low-density lipoprotein receptor) KO, deletion of PHD1 [105] or PHD inhibition [106] resulted to reduced atherosclerotic plaque development. 

On the other hand, genetic deletion of PHD2 in endothelial and hematopoietic mouse cells induced severe pulmonary vascular remodeling and right ventricular hypertrophy, characteristic features of clinical pulmonary arterial hypertension [107]. Although the phenotypes caused by PHD KO cannot be necessarily attributed to HIF activity, since PHDs may also have additional substrates or partners [108], pulmonary hypertension has been long known to be linked to HIF activation, since exposure to chronic hypoxia can indeed cause pulmonary arterial smooth muscle cell proliferation, migration and hypertrophy leading to pulmonary vascular remodeling and eventually pulmonary hypertension [109]. Many studies with both human subjects and animal models have implicated HIFs in the response of the pulmonary vasculature to hypoxia and also revealed the involvement of HIFs in forms of pulmonary hypertension not directly caused by hypoxia (reviewed in [110]). Pulmonary vascular remodeling is supported by extensive metabolic reprogramming, affecting both glucose and lipid metabolism, many aspects of which may be mediated by HIFs [111,112]. The importance of this reprogramming is illustrated by the fact that deficiency of malonyl-CoA decarboxylase, a key regulatory enzyme for fatty acid oxidation, in mice can attenuate the vasoconstriction and vascular remodeling caused by hypoxia [113,114]. Recent metabolomics studies in a murine model of pulmonary arterial hypertension have indeed shown changes in lung tissue lipid composition compatible with HIF-dependent metabolic reprogramming [115]. However, whether any of the HIF targets listed Table 1 is directly involved in pulmonary vascular remodeling remains to be shown. 

## 6. Conclusions

Recent information gathered from investigations in cell lines, animals and patient biopsy samples signify the importance of hypoxia and HIF activation in the regulation of lipid metabolism, and their contribution to the development and progression of cancer and other pathological conditions associated with the accumulation of lipids in various types of cells and organs. A number of HIF inhibitors are currently being tested, along with conventional therapies, for the treatment of different types of cancer [116], as cancer cells depend on HIF function, including HIF-mediated stimulation of lipid synthesis, for survival, proliferation and metastasis. As many studies have also shown that HIF inactivation by deletion, silencing or chemical inhibition can revert the effects of lipid accumulation in various mouse models, targeting of HIF function may also represent a valid therapeutic approach in metabolic diseases. However, as the repertoire of direct HIF targets involved in the complex regulation of lipid metabolism is far from exhausted, further detailed investigation is required to reveal the exact steps controlled by HIFs, especially in terms of HIF-α isoform and tissue specificity.

## Figures and Tables

**Table 1:** Representative HIF direct or indirect target genes that mediate reprogramming of lipid metabolism under hypoxia.

Functional Category /Protein Name	HIF Isoform & Effect	Outcome & Experimental Evidence	Ref.
**FA & Lipoprotein Uptake**			
**PPARγ**	HIF-1 Positive	Increased expression HIF-1 binds to the promoter of *PPARγ* and activates its transcription	[48]
**FABP3**	HIF-1 Positive	Increased expression HIF-1α depletion inhibits the induction of *FABP3* under hypoxia	[49]
**FABP4**	HIF-1 Positive	Increased expression HIF-1 binds to the promoter of *FABP4* and activates its transcription	[50]
**FABP7**	HIF-1 Positive	Increased expression HIF-1α depletion inhibits the induction of *FABP7* under hypoxia	[49]
**LRP1**	HIF-1 Positive	Increased expression HIF-1α binds to the *LRP1* promoter and activates its transcription	[51]
**VDLR**	HIF-1 Positive	Increased expression HIF-1α depletion inhibits activation of VDLR promoter under hypoxia	[52]
**Reductive Carboxylation of Glutamine**			
**GLS1**	HIF-1 Positive	Increased expression HIF-1α depletion inhibits the induction of *GLS1* under hypoxia	[58]
**OGDH2**	HIF-1 Negative	Increased proteolysis SIAH2 (a HIF-1 target) mediates proteolysis of OGDH2	[57]
**Ceramide Salvage**			
**NEU3**	HIF-2 Positive	Increased expression HIF-2α binds to the *NEU3* promoter and activates its transcription	[101]
**FA Synthesis**			
**SREBP-1**	HIF-1 Positive	Up-regulation Inhibition of HIF-1 impairs phospho-SREBP-1 increase under hypoxia	[59,69]
**FASN**	HIF-1 Positive	Increased expression Inhibition of HIF-1 impairs the induction of FASN under hypoxia Increased binding of SREBP-1 to the FASN promoter under hypoxia	[59]
**TG Synthesis**			
**AGPAT2**	HIF-1 Positive	Increased expression HIF-1 binds to the promoter of *AGPAT2* and activates its transcription	[62]
**Lipin-1**	HIF-1 Positive	Increased expression HIF-1 binds to the promoter of *LPIN1* and activates its transcription	[63]
**LD Accumulation**			
**PLIN2**	HIF-2 Positive	Increased expression HIF-2α depletion inhibits the induction of *PLIN2* under hypoxia	[65]
**HIG2**	HIF-1 Positive	Increased expression HIF-1 binds to the promoter of *HIG2* and activates its transcription	[66]
**β-Oxidation**			
**PGC-1α**	HIF-1 & HIF-2 Negative	Reduced expression HIF-1α or HIF-2α depletion inhibits reduction of PGC-1α expression under hypoxia	[69]
**CPT1A**	HIF-1 & HIF-2 Negative	Reduced expression HIF-1α or HIF-2α depletion inhibit reduction of CPT1A expression under hypoxia	[69,70]
**MCAD**	HIF-1 Negative	Reduced expression HIF-1α depletion inhibits reduction of MCAD expression under hypoxia	[71]
**LCAD**	HIF-1 Negative	Reduced expression HIF-1α depletion inhibits reduction of LCAD expression under hypoxia	[71]
**PGC-1β**	HIF-1 Negative	Reduced expression HIF-1α depletion inhibits reduction of PGC-1β expression under hypoxia	[71]
